# Testing a counseling message for increasing uptake of self-injectable contraception in southern Malawi: A mixed-methods, clustered randomized controlled study

**DOI:** 10.1371/journal.pone.0275986

**Published:** 2022-10-18

**Authors:** Holly M. Burke, Catherine Packer, Akuzike Zingani, Philemon Moses, Alissa Bernholc, Lucy W. Ruderman, Andres Martinez, Mario Chen

**Affiliations:** 1 FHI 360, Durham, North Carolina, United States of America; 2 Centre for Health, Agriculture Development Research and Consulting (CHAD), Blantyre, Malawi; KEMRI-Wellcome Research Program, KENYA

## Abstract

**Objective:**

While self-injection of subcutaneous depot medroxyprogesterone acetate (DMPA-SC) has well-documented benefits, uptake may be improved by addressing client concerns such as fear of self-injury and low self-efficacy. However, current training materials for family planning providers do not address these concerns. We used an iterative process with family planning providers and clients, male community leaders and partners, and stakeholders in Malawi to develop a counseling message addressing user-centered concerns about self-injection. We report on our testing of the effectiveness of this evidence-based message for increasing self-injection uptake in the context of full method choice.

**Methods:**

We randomized 60 public facilities across two districts in southern Malawi to orient their providers to the message (treatment) or not (control). After strengthening data quality, we extracted routine service delivery data from the facilities six months before and after introducing the message. We compared pre- and post-orientation trends for the treatment and control groups using generalized linear mixed models. We conducted eight focus group discussions with a sample of providers oriented to the message.

**Results:**

The message was feasible to implement and highly acceptable to providers. During June 2020–June 2021, 16,593 new clients used injectables in Mangochi district (52% DMPA-SC; 15% self-injected). In Thyolo district, 7,761 new clients used injectables during July 2020–July 2021 (29% DMPA-SC; 14% self-injected). We observed high variability in number of clients and self-injection uptake across facilities and over time, indicating inconsistent offering of self-injection. In both districts, we found significant increases in self-injection in treatment facilities after message introduction. However, this increase was not sustained, especially when DMPA-SC was unavailable or about to expire.

**Conclusion:**

Based on the study findings, we recommend the evidence-based message be used in programs offering DMPA-SC self-injection services. However, effective use of the message is contingent upon a consistent supply of DMPA-SC.

## Introduction

Innovations in contraception provide users better options and allow greater opportunity to choose the method that best fits their desires and circumstances. One such innovation is subcutaneous depot medroxyprogesterone acetate (DMPA-SC), a self-injectable contraceptive that is packaged in a prefilled, easy-to-inject unit and provides three months of pregnancy prevention. Self-injection of DMPA-SC has been lauded as a game-changing option in the contraception method mix because of its potential to be more feasible and acceptable for users than existing options [1].

DMPA-SC self-injection is safe, efficacious, and acceptable to users, and evidence suggests that introduction and provision of this method can equal or improve contraception use compared to provider-administered injectables [2, 3]. While contraception preferences vary, self-injection has been shown to be acceptable for both adolescents and covert users [4–6], two groups that often encounter barriers to accessing contraception. Not only is self-injection acceptable for uptake, but continuation rates among self-injectors are routinely higher than those receiving DMPA-SC injections from providers [2, 7], indicating the potential for DMPA-SC self-injection to increase both uptake and continuation of contraception, particularly among hard-to-reach populations.

In sub-Saharan Africa, DMPA-SC use is increasing. For example, the percentage of all women using DMPA-SC nearly doubled in Uganda from 2017 to 2018 (from 4.5% to 8.8%) and in Burkina Faso between 2016 and 2020 (from 4.5% to 9.7%) [8]. However, these data were not further disaggregated into provider-injected and self-injection, and many countries do not disaggregate by subcutaneous and intramuscular (DMPA-IM) injection [9]. PMA2020, one of the only sources that separates subcutaneous and intramuscular injection, currently does not collect data in Malawi [10]. From 2015–2017 we conducted a randomized controlled trial in Malawi and found significantly higher continuation rates among women who self-injected DMPA-SC compared to those who received provider-injected DMPA-SC [3]. In 2018, about eight months after the trial, the Malawi Ministry of Health rapidly scaled-up self-injection [11]; however, since then, unpublished Malawi service delivery data have trended toward DMPA-SC self-injection uptake that was lower than anticipated.

There are several barriers to self-injection uptake, including potential users’ fear of injury, side effects, low self-efficacy, and partner objection [12–14]. For adolescents, additional reasons for not self-injecting relate to lack of access and sociocultural norms inhibiting adolescent use of self-injection, such as provider perceptions that adolescents cannot properly self-inject, misperceptions about the impact of injectables on fertility, and the conviction that adolescents should not be using contraception at all [6]. Privacy is a concern for both covert and adolescent family planning users, who may fear discovery of DMPA-SC units in their homes [5].

Despite documented concerns about self-injection, current training materials for providers neither include comprehensive guidance to present self-injection to clients nor take a user-centered approach. Training materials and job aids focus on teaching providers how to inject clients and outline key clinical information to deliver when explaining self-injection, such as storage and disposal of units, self-injection techniques, calculating reinjection dates, and side effects [15–18]. This information is sometimes provided in leaflets for self-injection users to take home [15]. Existing training modules and self-injection leaflets do not focus on user concerns about self-injection.

The primary objective of our study was to develop counseling messages about DMPA-SC and self-injection for family planning providers to address the concerns of potential users. Our secondary objectives included exploring barriers and challenges for consistent delivery of self-injection services, assessing the acceptability and feasibility of the delivery of the new messages, and testing the effectiveness of the messages for increasing self-injection uptake within the context of full method choice. In this paper, we focus on the secondary objectives.

Given that Malawi is not alone in seeking to increase uptake of this new method, our results provide timely and evidence-based guidance to Malawi and other countries offering or planning to offer DMPA-SC self-injection.

### Counseling message development

Our study consisted of two phases corresponding to the primary objective (“message-development phase”) and secondary objectives (“testing phase”). As the testing phase necessarily built upon the message-development phase, we summarize message development here.

To develop the message for testing, from May through December 2020, we used qualitative research methods in an iterative and collaborative process with our study partners in Malawi: Reproductive Health Directorate and Health Education Services of the Ministry of Health, Banja La Mtsogolo (BLM, an affiliate of Marie Stopes International), and Population Services International (PSI), as members of the national working group supporting scale-up of DMPA-SC.

The research was conducted in Blantyre, and Mangochi and Thyolo districts in southern Malawi. We drafted two messages in English and Chichewa which we assessed in Chichewa during eight focus group discussions (FGDs) with 64 family planning providers with experience counseling clients on self-injection in the public and private sectors. Providers from four service delivery channels were included: public sector facility-based health workers, public sector community-based health workers called health surveillance assistants (HSAs), private sector providers in BLM clinics in Mangochi and Thyolo, and staff from private pharmacies supported by PSI in Blantyre.

We refined the messages based on the rapidly analyzed provider data. The study team’s understanding from previous observations of self-injection service provision was that providers typically presented DMPA-SC as an alternative or replacement for DMPA-IM. However, the providers in the FGDs said they were already presenting DMPA-SC to clients as a self-injectable method, with the goal of having clients choose to self-inject—but we also learned they were delivering inconsistent information and not to all clients systematically. For example, during counseling some providers mentioned that diabetic patients in the community self-inject insulin without any problems or showed clients images of or actual DMPA-SC and/or IM injection units. Providers did not view the need for different counseling messages in the various service delivery channels, as they were concerned that different messages could confuse women, and therefore recommended that the same message be used in all channels.

After working with our study partners to refine and reduce the messages into a single message, we assessed that version in Chichewa in interviews with 30 adult, female family planning clients (of HSAs, public and private sector facilities, private pharmacies) who had never used DMPA-SC and in four FGDs with 37 male community leaders and male partners of DMPA-SC users in Mangochi and Thyolo.

Rapid analysis of these data from all participant types showed fear as the primary barrier of self-injection. Several types of fear were described: mostly fear that women would not self-inject properly; fear because it is a new method, fear of side effects, and fear of pain from the needle. Most family planning clients interviewed knew very little about DMPA-SC and self-injection and, for some, the study was their first-time hearing about it. Providers also mentioned low community-level awareness of the method. In contrast, most of the men knew about DMPA-SC but that is because we recruited men whose partners use DMPA-SC and we also recruited male community leaders, some whose wives used DMPA-SC. Despite having more familiarity with the method, male participants and providers described rumors or misperceptions in the community including that DMPA-SC caused infertility, was fatal or harmful, caused side effects like prolonged bleeding, and decreased men’s virility.

**Fig 1:**
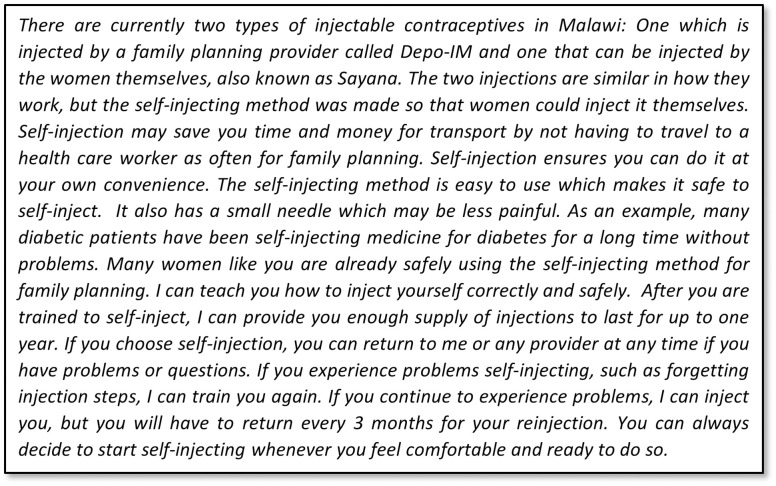
Evidence-based message used in testing phase (English version).

In collaboration with our study partners, we finalized the message to be tested in the next study phase (Fig 1) based on the qualitative research findings and stakeholder input. We addressed the multiple aspects of women’s fear of self-injecting in different parts of the message. First, we stated that DMPA-SC was designed so that women could self-inject, and we assured women that self-injection is easy and safe. Second, to normalize self-injection, we likened it to an example with which people in Malawi were already familiar—that is, diabetic patients have been self-injecting insulin for a long time—and asserted that other women were already self-injecting DMPA-SC. To address fear of incorrect self-injection, we explained that the provider would teach them how to self-inject correctly and safely, and we had providers explicitly offer ongoing support if women should encounter any problems. As providers felt strongly about emphasizing the advantages of self-injecting over provider-administration, we articulated the most salient benefits from our research findings: self-injection saves time, offers convenience, and has a smaller needle that may cause less pain. Finally, emulating several providers’ current practices, the message stated that if the client did not feel ready to self-inject today, she could do so next time or when she felt ready.

## Methods

### Design and overview

We used quantitative and qualitative research methods to test the effectiveness of the message on self-injection uptake. For the quantitative research, we conducted a clustered randomized controlled study in Mangochi and Thyolo districts. We randomized 60 public sector facilities to receive orientation on the message (treatment group) or to receive no message (control group). We compared trends in self-injection uptake among first time family planning users who used injectables using de-identified, routine service delivery data from the District Health Information Software 2 (DHIS2) [19] from six months before and six months after the providers were oriented to the message for the treatment and control groups. Because we sought to test the effectiveness the message on self-injection uptake and wanted to exclude clients who might have been exposed to previous counseling around self-injection, we extracted data only from first time family planning users who received injectables: DMPA-IM, provider-administered DMPA-SC, and DMPA-SC self-injection.

Study staff conducted monthly in-person data strengthening visits in the study facilities (treatment and control). The purpose was to work with providers and data clerks to identify and resolve data quality issues for the DHIS2 data. During these visits we also collected information about non-data issues which significantly impacted delivery of DMPA-SC and self-injection services in the two districts.

For the qualitative research, we conducted FGDs with a sample of providers oriented to the message, four to six months after their orientation to learn if and how they had used the message, their perceptions of its effect on uptake of self-injection, and their feedback on the message.

### Sample selection and randomization

Our goal was to include 30 public sector facilities in each district which were providing free family planning services to communities and reporting into DHIS2 to conduct the randomized study. In May 2020, we generated lists of all public facilities in each district using DHIS2: 32 facilities in Mangochi and 38 facilities in Thyolo. We then excluded facilities that would complicate data analysis because they included data from BLM (one facility in each district) or served the fewest DMPA-SC clients (based on data from October 2019 through March 2020) and selected 30 facilities per district. In November 2020, after stratifying the facilities based on number of new family planning clients they served (low, medium, and high) based on data from June through September 2020, we randomized 15 facilities in each district to the treatment group and 15 to the control group. The providers selected for orientation to the message were all HSAs and facility-based providers who provide DMPA-SC and self-injection and are affiliated with the facilities randomized to the treatment group.

The sample size for the quantitative research was based on feasibility considerations. However, based on preliminary data for the population of new family planning clients, we estimated having adequate power (83%) to detect a 23% difference in the uptake of self-injection from a baseline uptake of 42% among new clients using DMPA-SC. For this power calculation we assumed 30 facilities per district, a balanced allocation of facilities into the treatment and control groups, an average of 100 new clients per facility in the six months after implementation, and an intraclass correlation of about 19%, which was estimated using earlier data. However, we acknowledge that preliminary data uncovered implementation barriers that should be addressed to allow us to test the impact of the intervention more conclusively.

We aimed to conduct eight FGDs with six to 10 public sector providers in each group because evidence indicates that 90% saturation of study themes can be reached within five FGDs [20]. After grouping the treatment facilities into four geographic zones, we randomly selected two zones per district in which to conduct discussions with HSAs and two zones per district for FGDs with facility-based providers. We then randomly selected eligible providers from all facilities within those zones. Eligibility criteria included: public sector provider in the treatment group who was oriented on the message, 18 years or older, had experience providing DMPA-SC and self-injection counseling, willing to provide written informed consent, and willing to be audio recorded during the FGD.

### Message orientation

**Fig 2:**
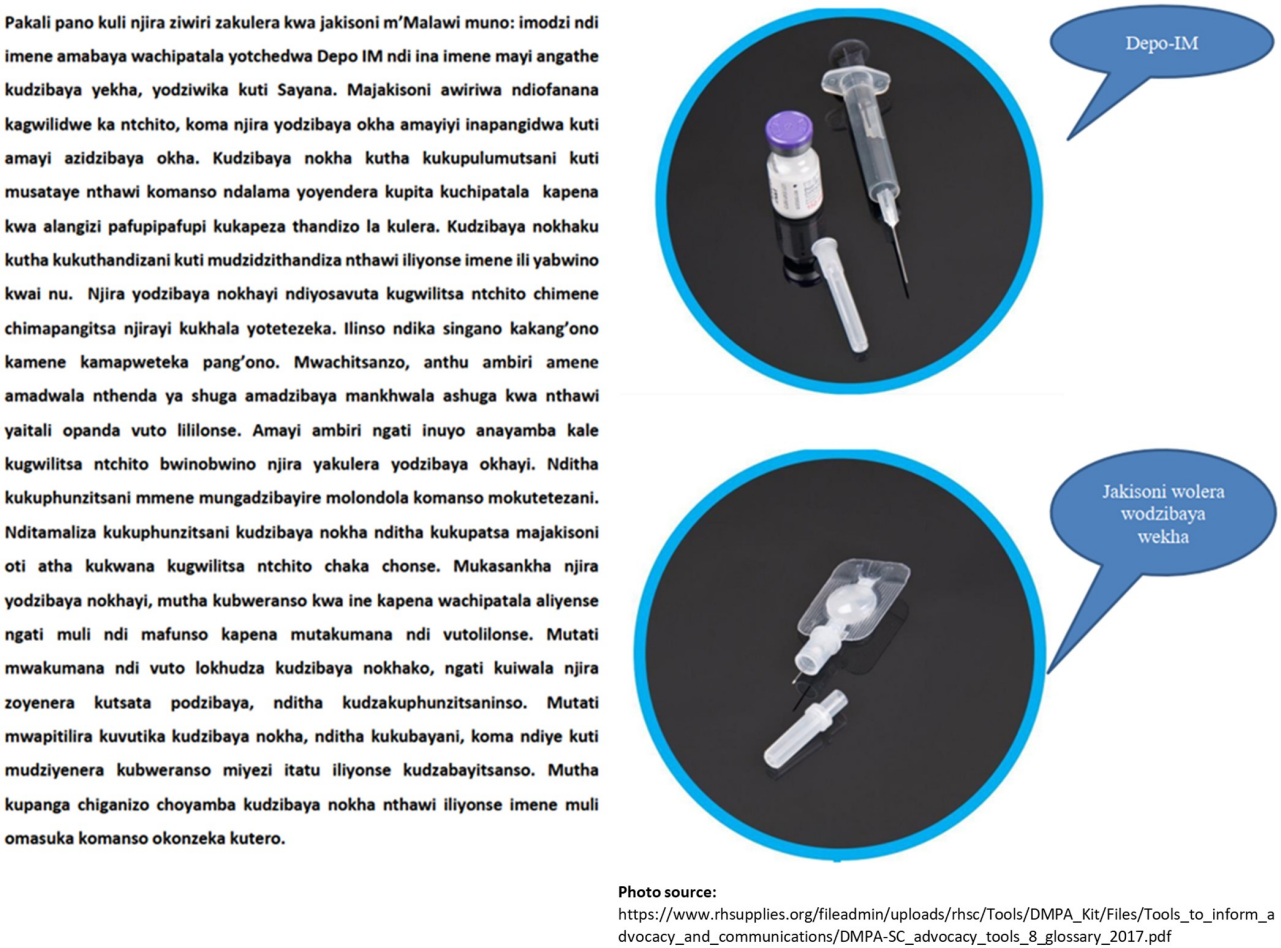
Evidence-based message used in testing phase, in Chichewa, with pictures of the two injectable options.

The Mangochi providers were oriented on the final message for testing in December 2020, and the Thyolo providers in January 2021. The provider orientation was designed in collaboration with each district family planning coordinator to mimic real-world implementation. In Mangochi, all providers were oriented during in-person sessions lasting approximately three to four hours which included role play to help providers determine how to best incorporate the message into their counseling. In Thyolo, we used a cascade approach where a subset of providers was oriented during an in-person session of around 90 minutes to two hours, and these providers were asked to cascade the information to the remaining providers. Instead of role plays, in the Thyolo orientations we asked providers to describe how they counseled and then suggested how to incorporate the message into their counseling. Each provider was given their own one-page, double-sided, laminated copy of pictures of DMPA-IM and DMPA-SC and the evidence-based message in Chichewa (Fig 2).

### Data collection

For the quantitative research, we extracted facility-level DHIS2 data monthly during June 2020– June 2021 for 30 facilities in Mangochi and July 2020–July 2021 for 30 facilities in Thyolo.

From January 2020 through August 2021, study staff conducted monthly in-person data-strengthening visits in all 60 study facilities, alternating between Mangochi and Thyolo districts. During these visits, we reviewed HSA and facility-based providers’ family planning registers and facility reporting forms to ensure the correct versions of the forms were being used and that they were being completed correctly. We identified the sources of errors whether at the provider, facility, or data clerk level. The scope of these visits increased over time as we learned about issues beyond data quality which significantly impacted delivery of DMPA-SC and self-injection services. In September 2020, we developed a tool to use during the visits to ask providers about DMPA-IM, DMPA-SC, and self-injection leaflet stock issues and other challenges to providing services such as imminent expiration of DMPA-SC, staffing shortages, and restrictions and other challenges related to the COVID-19 pandemic. Information about stock was not collected every month or from every provider, therefore, we did not systematically assess stock outs and instead use the term “stock issues” in reference to these data.

Using a semi-structured FGD guide, trained moderators conducted four FGDs in Mangochi in April 2021 and four in Thyolo in June and July 2021. Due to stock outs of DMPA-SC in Thyolo district, we delayed the FGDs in Thyolo until providers would have a chance to use the message. After providing written informed consent, participants completed a short demographic survey to collect age, sex, and experience providing family planning services, including counseling on DMPA-SC and self-injection using KoBoToolbox software [21]. FGDs were audio recorded and then transcribed and translated verbatim into English transcripts.

### Data analysis

For the quantitative research, we extracted the information from the monthly facility-based Excel files into SAS [22] and created a dataset from which we graphed and summarized the DHIS2 data. Given the different service delivery contexts and supply constraints vary, as well as the Ministry of Health’s preference, analyses for each district were done separately. We described trends in self-injection uptake among new family planning clients who used injectables over time for the treatment and control groups and compared pre- and post-orientation trends to assess the effectiveness of the message in the treatment facilities. We used a generalized segmented linear mixed regression model with a logit link to model the likelihood of using self-injection (0 = No, 1 = Yes) among all new injectable users. The model included a treatment group indicator, pre- and post-orientation status (month of the message orientation as the main point of interruption), time (in months) and interactions, and accounted for the nesting of individuals within facilities using random effects. We used this model to see if there were changes in trends (slopes) before and after the orientation and differences in these trends between treatment and control groups. We also assessed whether there was an immediate effect of the message at the beginning of the treatment period. This is modelled as a jump in the likelihood of self-injection immediately after the orientation, setting up a potential new trend. Because of the logit link, we present these estimates of changes in slopes and jumps as odds ratios with 95% confidence intervals. Furthermore, using the model estimates, we predicted the probability of self-injection and computed the mean of these probabilities to compare them with the observed proportion of self-injection over time. To ease the visualization of these trends, we plot linear approximations.

Information about stock issues obtained from the data-strengthening visits are included in the graphs to provide context to the interpretation of the model results.

For the qualitative research, we summarized the participant demographic survey data descriptively using Excel. The data from each transcript were summarized in an Excel rapid analysis matrix [23, 24] based on the FGD guide and preliminary review of transcripts. Two analysts independently completed the first matrix and discussed responses to ensure consistency, and then one analyst completed the remaining seven transcripts. Lastly, the themes were summarized into memos using the matrices.

### Ethics statement

This study was reviewed and approved by Malawi’s National Health Sciences Research Committee, FHI 360’s Protection of Human Subjects Committee, and MSI’s Ethics Review Committee. All qualitative research participants signed informed consent forms prior to participating in an FGD.

## Results

To contextualize the quantitative trend findings, we present the qualitative and data strengthening findings first.

### Focus group discussions with providers

Seventy-five providers were invited to take part in an FGD, eight of whom were unavailable on the scheduled date and one of whom was replaced. The resulting 68 providers who participated came from 26 out of the 30 intervention facilities (14 in Mangochi, 12 in Thyolo).

Slightly more than half (53%) of the participants were male, and their average age was 38 years. Almost all participants had been offering provider-administered DMPA-SC (94%) and self-injection (93%) for more than one year. Participants estimated having ever counseled an average of 689 clients on self-injection and training 230 clients to self-inject. All providers in the FGDs reported using the message with at least three clients, and the average was 90 clients.

#### Acceptability of message

Overall, both HSA and facility-based provider participants reported positive experiences delivering the message. Participants liked the message and said it was different from how they previously talked with women about DMPA-SC and self-injection. They appreciated having a uniform message with straightforward information, as described by this facility-based provider from Thyolo:

“*In the past*, *we were just sharing about Sayana depending on what we remembered from training*. *Because we didn’t have a guide… While now*, *the message is uniform*. *So whatever message I share*, *my colleagues are sharing the same message across facilities*.*”*

#### Feasibility of message

Participants from all FGDs said they thought it was not only feasible to train all providers on the message, but that doing so was important to ensure that clients are getting the same information. Some participants felt the message was easier to deliver to clients because the information was presented more clearly compared to how they used to counsel. One HSA in Mangochi explained, “*Since I started using this message*, *it has been a simple way reaching a lot of people at once during counseling*.*”*

Most providers said they read the message directly from the card given to them during the orientation. Some said they used to read it but had memorized it and still used the card as a guide and to show clients the pictures. Regardless of whether they read or had memorized the message, most providers said they delivered it as written and had not changed any parts of the message. Overall, participants felt that other providers would use the message as written if they were trained to use the message.

#### Use of message

Participants described a range of situations when they used the message, most frequently in group and individual counseling, as well as at village health talks, home visits, under-five clinics, and antenatal care visits. Most participants said they used the same message with new and returning clients, irrespective of prior DMPA-IM use, age, and covert or overt family planning use. There were some exceptions. For example, several providers said that although they used the same message, they provided additional details to new clients, who typically have more questions or know less about family planning. Some providers described emphasizing the benefits of DMPA-SC such as smaller needle size the ability to save time by self-injecting, particularly with previous users of DMPA-IM. Many participants mentioned that DMPA-SC stock issues affected their ability to use the message, as this provider describes:

*“I am still also using this message to counsel women about Sayana*. *Though the current challenge we have now is that the current stock that we have is expiring soon*, *so this wouldn’t be applicable to some part of the message where we are assuring them that we can offer them a yearlong supply while our current stock is expiring soon*.*”–Facility-based provider*, *Mangochi*

#### Potential impact of message

When asked if they thought the message influenced whether clients chose self-injection or provider-administered DMPA-SC, most providers in all focus groups felt that the message influenced women to choose self-injection, as illustrated by an HSA in Mangochi:

*“Since I started giving counseling using the message card*, *it seems 70% of women have started using this method of self-injecting… This resource has helped a lot in enticing women to start using this method*.*”*

The main reasons they perceived for why clients chose self-injection related to the message’s emphasis on its benefits vs. provider-administered DMPA-SC and DMPA-IM, especially the ability to take home a year’s supply and the need to return to a provider less frequently, saving them time and money. As an HSA in Thyolo explained,

*“This message motivated the women to choose this method of Sayana to inject themselves*, *because there are good reasons in this message*, *the likes of reducing transport costs for reaching to a clinic*, *the likes of reducing time to go meet a counselor*, *in so doing they are saving time*.*”*

Other reasons included self-injection being good for discreet family planning users because women can use it privately at their convenience; the smaller needle, which often came up when providers showed women the picture of DMPA-SC and DMPA-IM; and the message’s example of diabetic patients safely self-injecting insulin, which providers said helped address women’s fears of self-injecting, as illustrated by this HSA from Mangochi:

*“The coming of this message has changed a lot of things*. *At first*, *we used not to explain in details and that’s the reason most women were not accessing Depo-SC*. *but with this message*, *especially with an example that was given inside of diabetic patients who self-inject their medication*, *women understood and wished there was a chance to get doses for the whole year*.*”*

#### Participant recommendations

We asked participants for recommendations about how to improve the message. The most common responses were the need for more copies of the counseling message. Some participants suggested shortening it, adding more pictures, or integrating it into the national family planning flip chart. Several participants also discussed ideas for monitoring, supervision, or mentorship to ensure that providers were supported and using the counseling card correctly. Providers in Mangochi suggested translating the message into Yao, another language spoken in the district. Some participants also requested an adequate supply of self-injection leaflets and family planning flip charts. Others mentioned that having additional materials such as posters or educational materials about DMPA-SC in facilities and communities would help women become more familiar with self-injection, which could be reinforced by all providers using the message. Finally, some participants said that having stock of DMPA-SC would help them consistently use the message.

### Insights from data-strengthening visits

We identified three common factors affecting self-injection uptake during the data-strengthening visits in both districts: (1) limited stocks of injectables, (2) expiring DMPA-SC stock, and (3) COVID-19.

Both districts experienced limited stocks of injectable contraceptives across all months. Due to inconsistent stock, providers reported that clients were losing faith in injectables and were switching to other methods. We also heard that when stock is low, providers are less likely to offer self-injection or may only give self-injectors one or two units to take away, thus limiting the primary benefit of self-injection. However, some stock issues may have been mitigated through some providers “borrowing” stock from other facilities to fill the needs of “loyal” clients. Finally, some HSAs were only given a small amount of stock and were dependent on the umbrella facility’s stock.

Imminent expiry was an issue when DMPA-SC stock was available. In some such cases, providers would only administer DMPA-IM, but in most cases, providers said they were encouraged to minimize DMPA-IM use and encourage DMPA-SC so that it could be used before it expired. However, providers could not give additional DMPA-SC doses to clients trained to self-inject because the units would expire before the clients were due for their next injections.

Concerns about COVID-19 made it difficult for facilities to conduct outreach and limited the amount of time providers could spend with clients. In some cases, facilities were enforcing mask restrictions despite a mask shortage, and clients without masks were turned away. In early 2021, rumors that the COVID-19 vaccine was in injectable contraceptives began making some clients who were distrustful of the COVID-19 vaccine fearful of opting for injectable methods.

### Public sector delivery data

From June 2020 through June 2021, 16,593 new family planning clients used injectables in the 30 public Mangochi facilities included in the study (Table 1). Just over half of those clients used DMPA-SC and 15% self-injected. In the 30 Thyolo public facilities included in the study, 7,761 new family planning clients used injectables from July 2020 through July 2021. Less than one-third of those clients used DMPA-SC and 14% self-injected.

**Table 1:** New family planning clients who used injectables, by district and injection type (Malawi DHIS2 data).

	Mangochi (Jun 2020-Jun 2021)^1^			Thyolo (Jul 2020-Jul 2021)^2^		
Injection Type	Treatment n (%)	Control n (%)	Total n (%)	Treatment n (%)	Control n (%)	Total n (%)
DMPA-IM	3001 (38.3)	4937 (56.4)	7938 (47.8)	2960 (70.9)	2516 (70.1)	5476 (70.6)
DMPA-SC PI	3198 (40.8)	2919 (33.4)	6117 (36.9)	699 (16.8)	464 (12.9)	1163 (15)
DMPA-SC SI	1643 (21)	895 (10.2)	2538 (15.3)	513 (12.3)	609 (17)	1122 (14.5)
Total	7842	8751	16593	4172	3589	7761

^1^One month of DHIS2 data from one facility was missing

^2^Two months of DHIS2 data from one facility was missing

**Fig 3:**
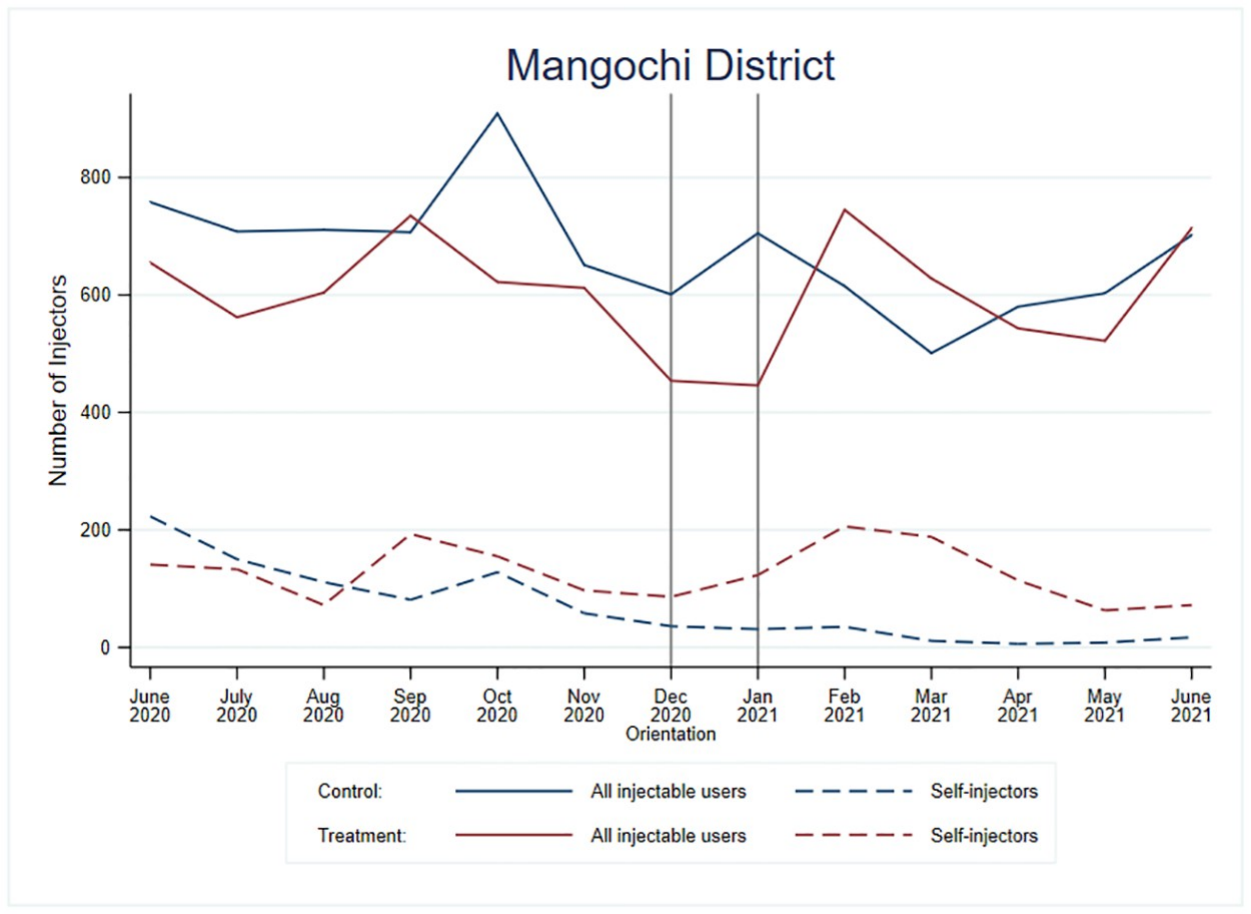
Number of total new injectable users and DMPA-SC self-injectors for treatment and control facilities in Mangochi District, Malawi.

**Fig 4:**
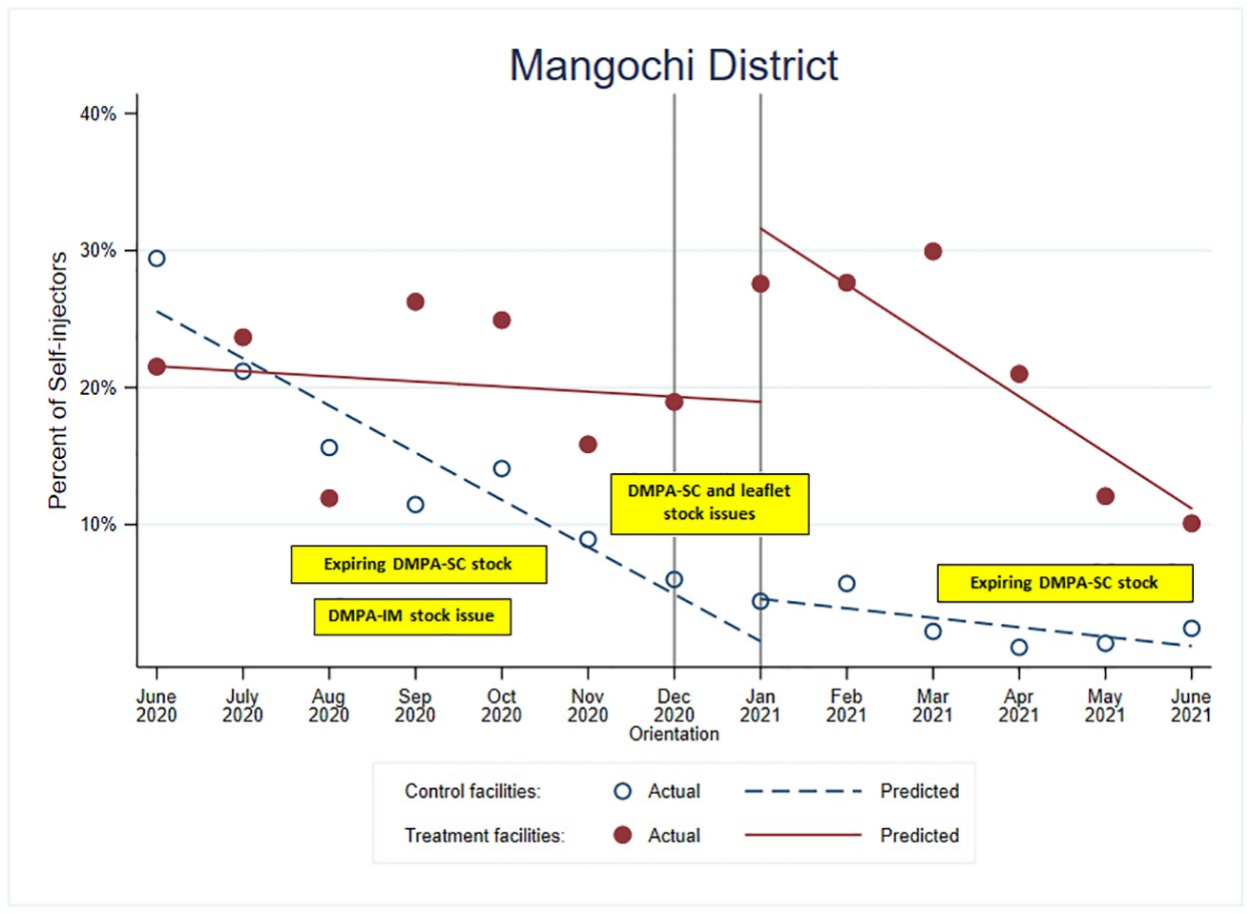
Percentage of total injections that were DMPA-SC self-injected and model-based trends for treatment and control facilities in Mangochi District, Malawi.

Figs 3 and 4 show the results for Mangochi district. In Mangochi district, there was high variability in the number of new family planning clients using injectables and self-injection uptake over time (Fig 3). Although not shown in the figure, there were also high variability across facilities.

Table 2 shows the estimated odds ratios for the trends before and after message orientation for treatment and control groups and the comparisons of these trends between groups (difference in difference). It also shows the estimate of the initial jump in the likelihood of self-injection after orientation in each group and the corresponding comparison. In Mangochi district, during the month following the message orientation, the change (jump) for those in the *control* facilities was not significant, but there was a significant *increase* for those in the treatment facilities. Comparing the change observed at this time point, the odds of self-injection in the treatment facilities increased almost four times more than that in the control facilities (OR = 3.78; p<0.01).

**Table 2:** Comparisons from model for treatment (T) and control (C) facilities in Mangochi and Thyolo Districts, Malawi.

	Mangochi		Thyolo	
Comparison	Odds Ratio Estimate (95% Confidence Interval)	P-value	Odds Ratio Estimate (95% Confidence Interval)	P-value
Trend pre-orientation (C)^1^	0.73 (0.70, 0.77)	<0.01	0.92 (0.87, 0.97)	<0.01
Trend pre-orientation (T)^1^	0.94 (0.90, 0.99)	0.01	0.82 (0.78, 0.88)	<0.01
Difference in trends pre-orientation (T vs. C)	1.28 (1.20, 1.37)	<0.01	0.90 (0.83, 0.98)	0.01
Trend post-orientation (C)^1^	0.75 (0.67, 0.84)	<0.01	0.76 (0.66, 0.88)	<0.01
Trend post-orientation (T)^1^	0.63 (0.60,0.67)	<0.01	0.88 (0.79,0.99)	0.03
Difference in trends post-orientation (T vs. C)	0.84 (0.74, 0.96)	0.01	1.16 (0.97, 1.39)	0.11
Difference in trends for C facilities (post vs. pre)	1.02 (0.90, 1.16)	0.71	0.83 (0.71, 0.97)	0.02
Differences in trends for T facilities (post vs. pre)	0.67 (0.62, 0.72)	<0.01	1.07 (0.94, 1.22)	0.28
Difference in difference in trends (T vs. C)	0.66 (0.57, 0.76)	<0.01	1.29 (1.06, 1.58)	0.01
Change (jump) after message orientation (C)	1.02 (0.70, 1.48)	0.93	0.45 (0.28, 0.70)	<0.01
Change (jump) after message orientation (T)	3.84 (2.97, 4.97)	<0.01	1.63 (1.02, 2.59)	0.04
Difference in jumps (T vs. C)	3.78 (2.40, 5.96)	<0.01	3.65 (1.90, 7.00)	<0.01

^1^ORs for trends (slopes) refer to comparisons of the odds of self-injection with each month increase

In Mangochi district, the trends in self-injection decreased over time for both groups and during the pre and post intervention periods, but the rate of decrease changed after intervention. After the initial large increase in the odds of self-injection in the treated facilities following message introduction, self-injection uptake decreased much faster in the treated facilities than in the control facilities (OR = 0.66; p<0.01). The odds of self-injection decreased for both groups over time; however, the proportion of self-injectors in the control facilities could not go much lower, and the odds of self-injection for the treatment facilities did not fall to the level of the control facilities during the study period. These trends and comparisons between groups are visualized in Fig 4.

In Fig 4, we overlaid the information we had learned about stocks of DMPA-IM, DMPA-SC, and the self-injection leaflet during the data-strengthening visits in Mangochi district with the model-based predicted trends to contextualize the results and suggest potential drivers behind the variability of self-injection uptake we observed. For example, the decreasing odds of self-injection for both groups may be explained by the stock issues experienced over much of the observation period. Specifically, during our data-strengthening visits in September 2020, we learned of significant stock issues of DMPA-IM and that the DMPA-SC stock would soon expire. In December, we learned that stock of DMPA-SC and leaflets had decreased. In February 2021, we learned that Mangochi had received re-distribution of DMPA-SC from Lilongwe, but the stock they had received would expire between April and November 2021, potentially increasing provider-administered DMPA-SC during that period because of the limited ability to provide multiple units of DMPA-SC to self-injectors for future use.

**Fig 5:**
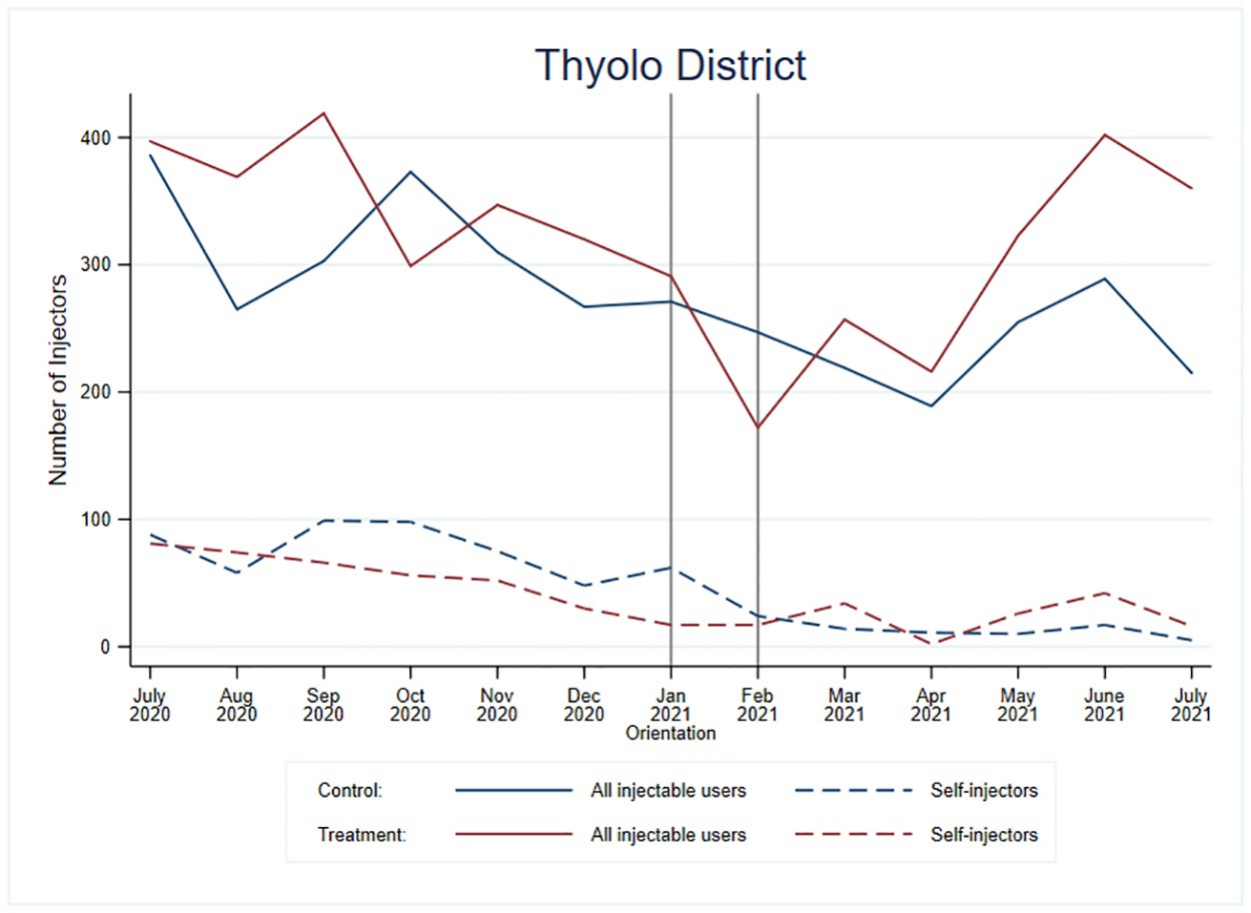
Number of total new injectable users and DMPA-SC self-injectors for treatment and control facilities in Thyolo District, Malawi.

**Fig 6:**
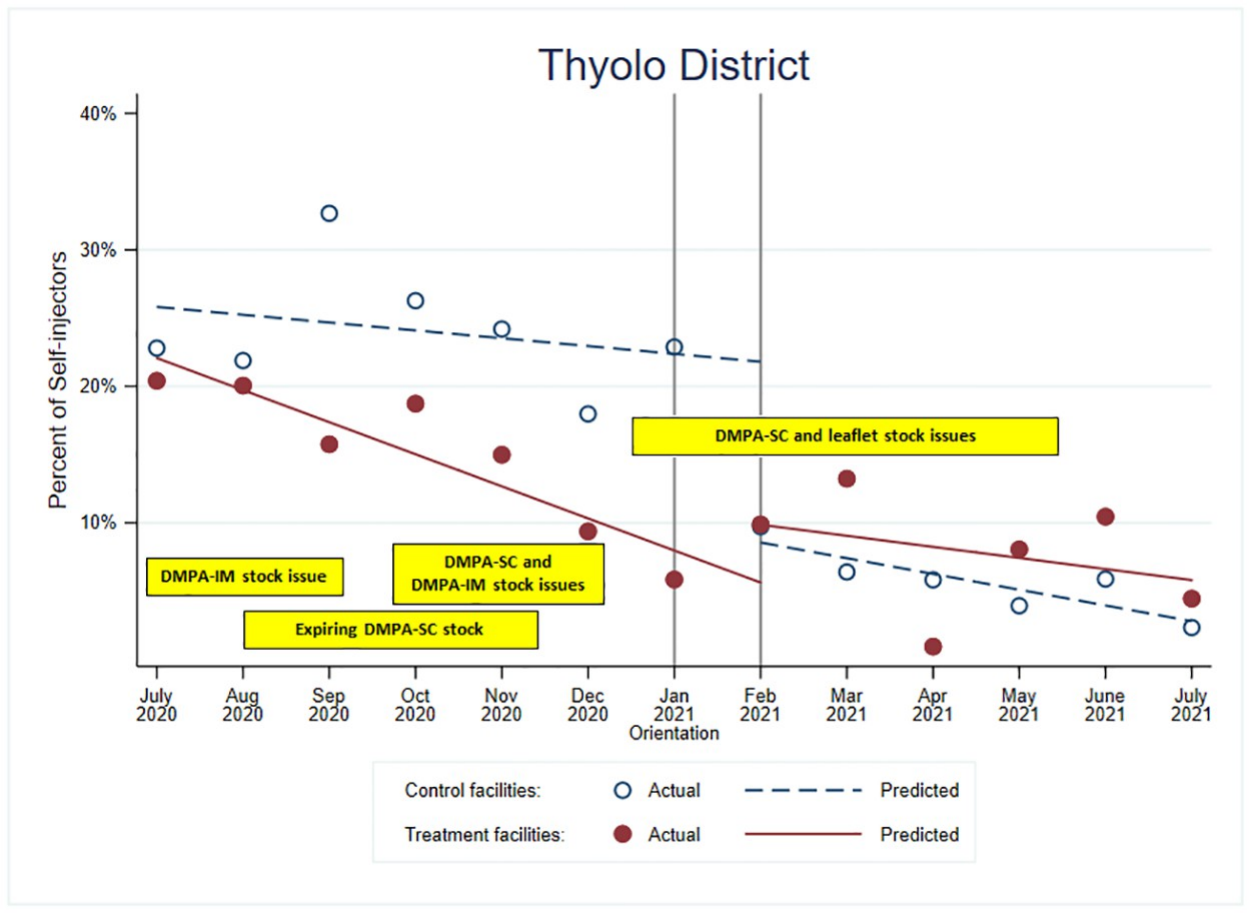
Percentage of total injections that were DMPA-SC self-injected and model-based trends for treatment and control facilities in Thyolo District, Malawi.

Figs 5 and 6 show the results for Thyolo district. In Thyolo district, there was high variability in the number of new family planning clients using injectables and self-injection uptake over time (Fig 5).

In Table 2, the jump in the odds of self-injection in Thyolo district was about four times greater for the treatment group than for the control group (OR = 3.65; p<0.01). The change for those in the *treatment* facilities was small, but positive, and there was a substantial *decrease* for those in the control facilities (Fig 6).

After orientation, the rate of decrease for the treatment group in Thyolo district was similar to its rate pre-orientation (OR = 1.07, p = 0.28), whereas the rate of decrease for the control group was significantly greater post-orientation than pre-orientation (OR = 0.83, p = 0.02). When comparing these changes in trends pre- to post-intervention between the groups, we observed an improvement in the trends in the treatment group as compared to the control group (OR = 1.29, p = 0.01) unlike to what we observed in Mangochi, although the trends are still downwards. Like Mangochi, the message appears to have prevented a similar decrease in the odds of self-injection for those in the treatment facilities.

We overlaid the data-strengthening visit information from Thyolo with the model predictions in Fig 6. We saw similar constraints as Mangochi; however, more Thyolo facilities reported having low or no stock, whereas Mangochi had had more expiring stock. In Thyolo, there were substantial DMPA-IM stock issues reported in July and August 2020. Like Mangochi, Thyolo had expiring DMPA-SC stock around September 2020, while around November 2020, stock issues were reported for both DMPA-IM and DMPA-SC in Thyolo. From January until around May 2021, stock issues were reported for DMPA-SC and self-injection leaflets, which corresponds to the drop we observed in DMPA-SC provision and self-injection uptake.

## Discussion

Self-administered injectable contraception is a self-care intervention that is recommended by the World Health Organization (WHO) [25]. While self-care interventions including DMPA-SC self-injection have the potential to increase choice and autonomy, they must be accessible, acceptable, and affordable. To address lower than anticipated uptake in self-injection in Malawi, we developed a counseling message addressing user-centered concerns about self-injection and tested the effectiveness of this message for increasing self-injection uptake in two districts in Malawi. We also explored the barriers and challenges for consistent delivery of self-injection services.

Through an iterative and collaborative message development process with family planning providers, clients, men, and stakeholders in Malawi, we learned that family planning providers were delivering inconsistent messages about self-injection with their current training. We also learned that fear of self-injection is a major barrier for women to try self-injection, highlighting an urgent need for counseling messages which explicitly help women overcome fear and increase their self-efficacy for self-injection. Malawi is likely not alone in this regard, since global trainings do not provide messages that address potential users’ concerns about self-injection.

Using routine service delivery data we observed significant increases in uptake of self-injection in facilities after their providers were oriented to the evidence-based message. However, this increase was not sustained over time, especially when there was limited availability of DMPA-SC or the stock was about to expire. Within this challenging service delivery context, the message appeared to act as a “shield” for the treatment facilities, preventing self-injection from decreasing and “preserving” uptake at about the same pre-orientation levels.

Importantly, we observed high variability in number of clients and self-injection uptake across facilities and over time, indicating that self-injection services were not being offered consistently in Mangochi and Thyolo districts. This inconsistency appears to be due to several factors, especially stock outs of DMPA-SC and self-injection leaflets, both of which greatly limit the ability of providers to offer self-injection to clients. It would be ideal to test the message in a context where client flow is stable and self-injection services are delivered consistently, since this would allow for a cleaner assessment of the magnitude of the message’s impact on self-injection uptake. However, we are not aware of any such ideal context.

### Strengths and limitations

This study is not without limitations. First, while private sector providers and clients were consulted during message development, we did not test the message in the private sector. We included three providers from one private clinic in each district during the public sector orientations, but the very low reported numbers of self-injectors in the two private clinics during the study period precluded us from being able to test the message in these clinics. Second, our analysis focused on new family planning clients who used injectables because we wanted to test a message that introduces DMPA-SC and self-injection to clients. Nonetheless, clients not new to injectables may also benefit from the message, and they should be included in future assessments of the message. In addition, our research was conducted in two districts in southern Malawi, but the influence of the message may differ in other settings. However, research in other countries has identified fear and low self-efficacy as barriers to self-injection uptake [12, 14, 26]—themes that are well-addressed by our message, suggesting that it may be beneficial in other settings.

While we worked to strengthen the quality of the DHIS2 data for DMPA-SC and self-injection in all treatment and control facilities, errors may still exist with these service data, which are collected by family planning providers and not researchers. Further, it is possible that the data from the beginning of the study had more errors than the data at the end of the study, though we attempted to minimize this by starting the data strengthening four months before extracting the data used in the analysis. Also, it is possible that data from clients served by other family planning outreach efforts may have been combined with the study facility data, but such outreach providers were not oriented on the message. Other factors that affect self-injection uptake may have impacted the facilities in different ways. For example, larger-volume facilities may have experienced less frequent stock outs than smaller facilities. However, the randomized design minimizes the differences across the treatment and control groups, and stratification of facilities prior to randomization minimizes potential bias from factors associated with client volume. Further, the use of routine service data minimized selection bias and offered a large sample size for the analysis. Finally, it is possible that social desirability bias may have influenced the FGD participants’ responses about acceptability of the message. We attempted to minimize this potential bias by having experienced moderators trained in qualitative research techniques conduct the discussions.

### Implications for practice

The first step in increasing uptake of self-injection in Malawi is to ensure a constant supply of DMPA-SC and self-injection leaflets to all facilities and providers, especially HSAs who serve communities. Based on the evidence this study generated, we recommend that the message be included in the standard DMPA-SC and self-injection provider training. It could be distributed during providers’ initial training, as well as during refreshers and supervision visits. The Malawi Ministry of Health may also want to consider incorporating the message into or alongside the national family planning flipchart, which provides an overview of all methods. Since DMPA-SC has recently been introduced, and self-injection is a new way to deliver family planning that requires new user actions and responsibilities, more information that addresses client concerns should accompany this method.

Other possible applications of the study’s findings include developing a visual presentation of the message for providers to show clients during counseling sessions or creating other communication materials based on the evidence-based message components. Additional pretesting of the images and format of these new materials would be needed with the intended audiences. Consistent with the High Impact Practice of engaging men in family planning [27], we also recommend developing informational materials targeting men and boys to combat negative rumors about DMPA-SC and self-injection and increase male support. Indeed, a recent analysis of population-based data from Burkina Faso, the Democratic Republic of Congo, Kenya and Nigeria found low use of and demand for self-injection; the authors called for more demand generation since they observed that only one country included in the analysis had promotional campaigns about self-injection [28]. However, before embarking on demand generation campaigns governments need to ensure there is sufficient supply to meet demand.

Finally, we recommend regular review of DHIS2 data for DMPA-SC and self-injection and providing support to strengthen these data. As we have been able to demonstrate within this study, accurate service delivery data can be used to examine trends and inform decision-making including procurement and distribution decisions which can ultimately ensure adequate stock of DMPA-SC.

## Conclusion

The evidence-based message that we developed was feasible to implement and resulted in significant increases in DMPA-SC self-injection uptake in two districts in Malawi. We recommend that programs offering DMPA-SC self-injection use this message and ensure a constant supply of DMPA-SC, which would allow the message to realize its full impact potential and may help with the sustainability of its effects.
